# Idiopathic granulomatous mastitis: a diagnostic dilemma for the breast radiologist

**DOI:** 10.1007/s13244-016-0497-2

**Published:** 2016-05-10

**Authors:** Smiti Sripathi, Anurag Ayachit, Archana Bala, Rajagopal Kadavigere, Sandeep Kumar

**Affiliations:** Department of Radiodiagnosis and Imaging, Kasturba Medical College, Manipal, Karnataka India

**Keywords:** Granulomatous, Mastitis, Ultrasound, Mammography, Histopathology

## Abstract

**Background:**

Idiopathic granulomatous mastitis is a chronic inflammatory disease of the breast, which is often difficult to differentiate both clinically and radiologically from infectious aetiologies such as tuberculosis, fungal infections, and also from malignancy, thus posing a diagnostic dilemma. We present a pictorial review of the commonly encountered imaging findings in idiopathic granulomatous mastitis on mammography and ultrasound.

**Materials and methods:**

Mammographic and ultrasound findings of histopathologically proven cases of granulomatous mastitis are discussed.

**Conclusion:**

Idiopathic granulomatous mastitis has varied and non-specific appearances on ultrasound and mammography. Histopathology is essential to establish diagnosis.

***Teaching Points*:**

• *Idiopathic granulomatous mastitis often poses a diagnostic dilemma for the radiologist by mimicking malignancy*.

• *It has varied and non-specific appearances on mammography and ultrasound*.

• *Histopathology is mandatory to establish the diagnosis and decide management*.

## Introduction

Idiopathic granulomatous mastitis is an uncommon chronic inflammatory condition of the breast of unknown aetiology, seen commonly in women of childbearing age, although peri-menopausal women may also be affected. The clinical and imaging diagnosis of this benign condition is often difficult as it can simulate many conditions including malignancy. Histopathology is essential to solve the dilemma and make a definitive diagnosis, thus avoiding unnecessary mastectomies. Therefore, adequate recognition of its radiological patterns is vital to differentiate it from malignancy.

## Epidemiology

Idiopathic granulomatous mastitis was first described by Kessler and Wolloch in 1972 [1]. Its true prevalence is unknown since it is often a diagnosis of exclusion. In a study by Baslaim et al., histopathologically proven cases of idiopathic granulomatous mastitis were found in 1.8 % of 1,106 women with benign breast disease. Although it is seen globally, a higher racial predilection in Latin and Asian women is known [2]. The diagnostic dilemma is because of its clinical and radiological picture, which is often non-specific and may mimic a malignant mass. The final diagnosis is confirmed by histopathology where there is non-necrotizing granulomatous inflammation of lobules [3]. As a breast radiologist, it is essential to be aware of the imaging features of this rare entity to prevent unnecessary mastectomies.

## Clinical presentation

The most common clinical presentation of this entity is a breast lump that may be of firm to hard consistency. Bilateral involvement is rare. Although the lump may be present in any quadrant, there is a tendency to involve the subareolar region, or there may be diffuse involvement of entire breast. The patient may also present with pain, erythema, swelling, or axillary lymphadenopathy [4] although inflammation may not always be present clinically, thus leading to misdiagnosis as a malignant lesion. Other chronic inflammatory conditions that should be considered in the differential diagnosis include plasma cell mastitis, tuberculosis, histoplasmosis, Wegener’s granulomatosis. [5].

## Etiopathogenesis

The exact aetiology is unknown and is controversial; however, response to steroids points towards an autoimmune origin and is the most widely accepted theory [6]. The association with lactation (up to 9 months after delivery) is explained by the extravasated lactational secretions (due to local trauma or infection) damaging the ductal epithelium and leading to a granulomatous inflammatory response [4]. Oral contraceptives can cause a chemically induced granulomatous reaction [7].

Duct ectasia, periductal mastitis complex: Non-puerperal mastitis may be seen in patients with underlying duct ectasia or cysts where chemical inflammation is produced because of cyst or duct rupture [8]. Later bacterial infection may also occur. In duct ectasia there is weakening of duct wall due to stasis of fatty inflammatory secretions, ductal dilatation, and duct wall rupture, leading to periductal chemical mastitis. Further necrosis and infection may lead to abscess formation, especially in peri-areolar region.

Inflammation and rupture of cysts can also cause focal chemical mastitis and abscess formation.

## Radiological features

### Mammography

Routine cranio-caudal (CC) and mediolateral oblique (MLO) views are obtained. Additional views such as spot compression and magnification views are also carried out as and when required.

**Fig. 1:**
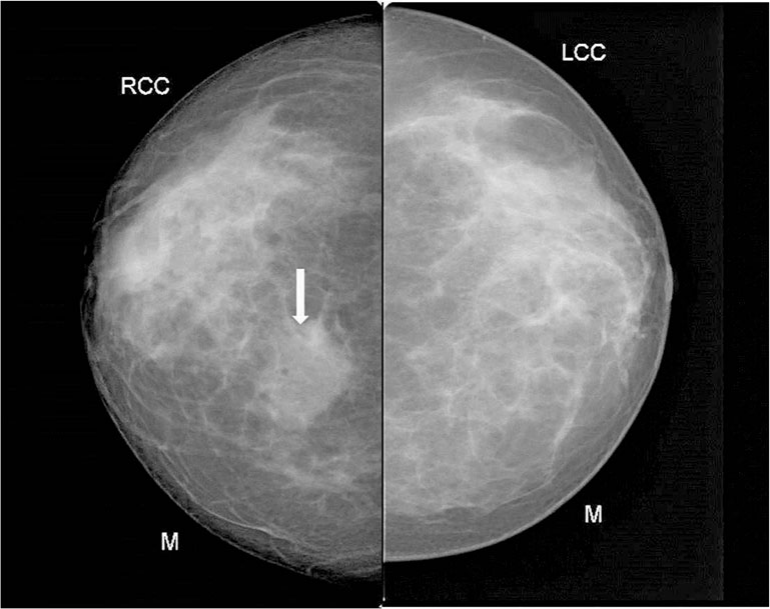
A 38-year-old woman presented with right breast lump of 3-week duration. Mammogram (cranio-caudal view) of the right breast shows an asymmetric opacity (arrow). Histopathology was s/o idiopathic granulomatous mastitis

**Fig. 2:**
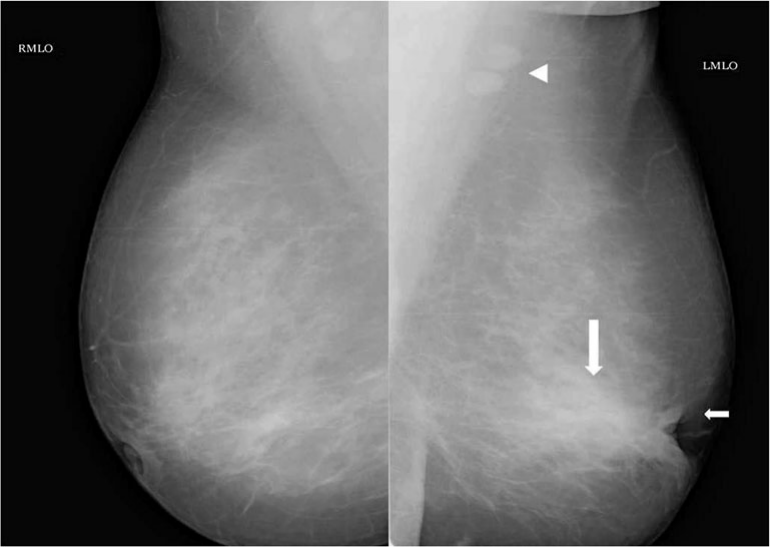
Mammogram (MLO view) of left breast of a 35-year-old woman presenting with a tender lump in left breast of 1-week duration revealed retraction of the left nipple (small arrow) with increased density in the retroareolar region (large arrow). A few benign axillary lymph nodes appearing enlarged were also seen (arrowhead). Histopathology from breast was suggestive of idiopathic granulomatous mastitis

**Fig. 3:**
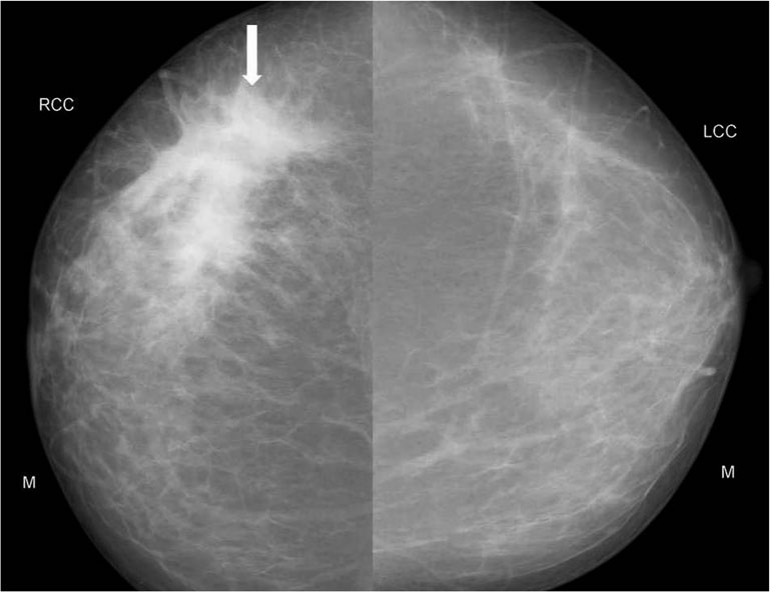
Mammogram (CC view) of a 30-year-old woman who presented with painless lump in the right breast of 20-day duration showed an ill-defined dense irregular opacity (arrow) with architectural distortion involving outer quadrant of right breast. A possibility of BIRADS category IV lesion was considered and biopsy of lump was done, which showed features of idiopathic granulomatous mastitis

Focal asymmetric density (Figs. 1, 2, and 3) is the most common mammographic pattern seen in idiopathic granulomatous mastitis, according to Yilmaz et al. [9] and Memis et al. [10]. Diffuse unilateral increase in breast density, more often seen in malignancies, may also be encountered. Mammograms of dense breasts may be reported negative since the findings cannot be appreciated well.

**Fig. 4:**
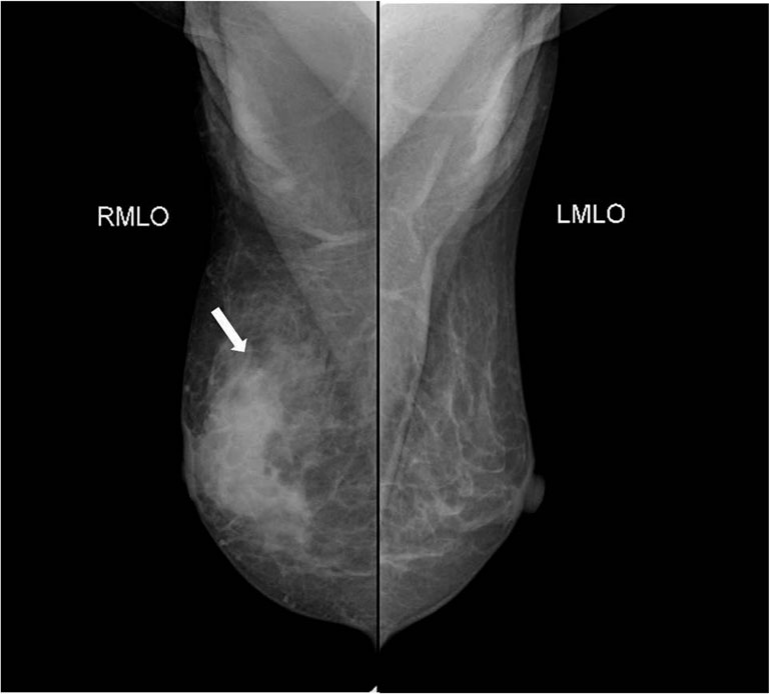
Mammogram (MLO view) of right breast in a 42-year-old woman presenting with progressively increasing lump in the right breast showed an ill-defined asymmetric opacity in the retroareolar region of the right breast (arrow) and right areolar skin thickening, compared to the opposite side

Han et al. [11] reported no visible changes in the skin or nipple areolar region in granulomatous mastitis; however, in a study by Lee et al. [12] overlying inflammatory skin thickening (Fig. 4) was demonstrated in 63.6 % of mammograms.

**Fig. 5:**
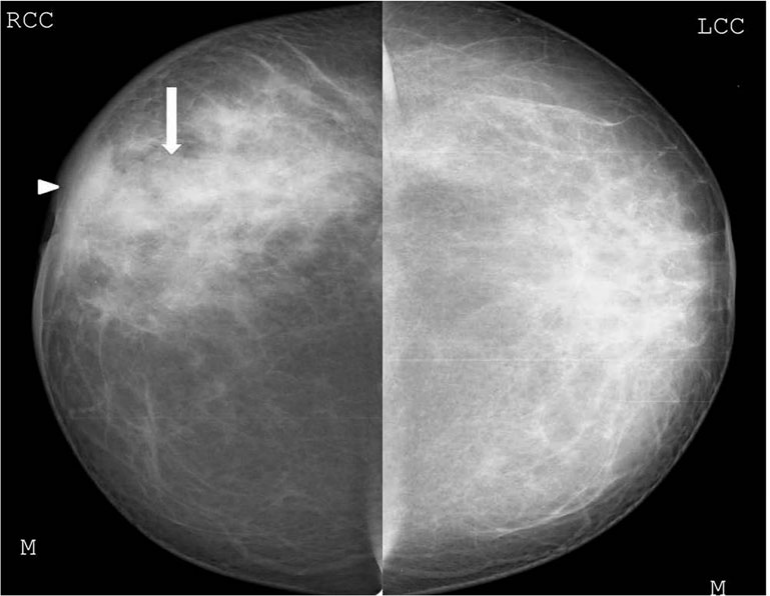
A 48-year-old woman presented with a tender lump of 2-week duration. There was no history of fever. Mammogram (cranio-caudal view) of right breast revealed increased density in retroareolar region (arrow) with overlying skin thickening (arrowhead). Histopathology was suggestive of granulomatous mastitis

Lee et al. [12] also described other associated features, including parenchymal distortion (Fig. 5), skin thickening, and benign appearing axillary lymph nodes with maintained fatty hila in 63.7, 63.7, and 54.5 % of subjects respectively.

**Fig. 6:**
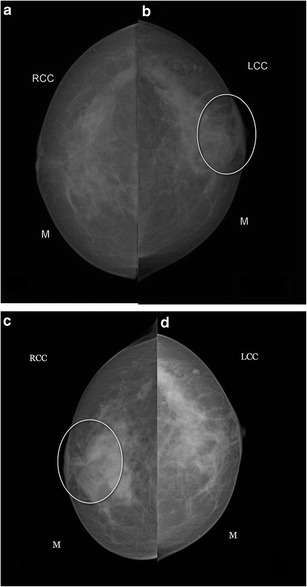
A middle-aged woman presented with swelling in the left breast associated with nipple discharge. (**a**, **b**) Mammogram (CC view) shows asymmetric breast density with skin thickening in nipple areolar region of the left breast. Histopathology was s/o. Granulomatous mastitis and patient was managed conservatively. The patient presented with swelling and pain in the right breast after 2 years with nipple discharge. Mammogram (**c**, **d**) revealed skin thickening and increased density in the right breast and was once again managed conservatively

Bilateral involvement is occasionally seen (Fig. 6a, b, c, d).

### Ultrasound imaging

**Fig. 7:**
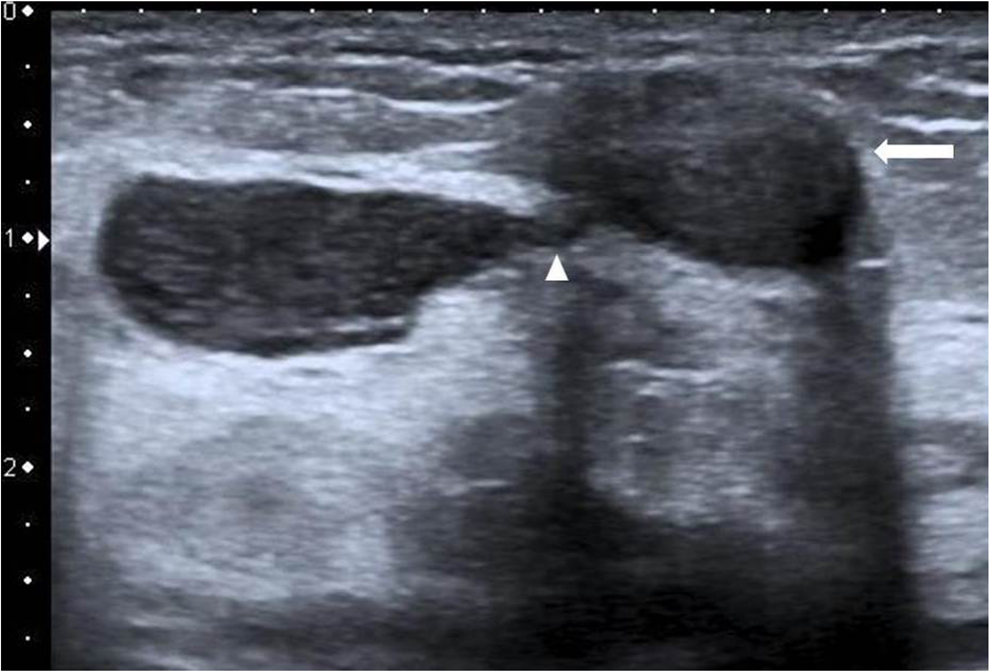
Ultrasound of the left breast in a 28-year-old woman who presented with breast lump and pain showed a few ill-defined hypoechoic lesions containing internal echoes (arrows) communicating with each other by tubular hypoechoic extensions (arrowhead). Biopsy was done under ultrasound guidance, and histopathology was suggestive of granulomatous mastitis

**Fig. 8:**
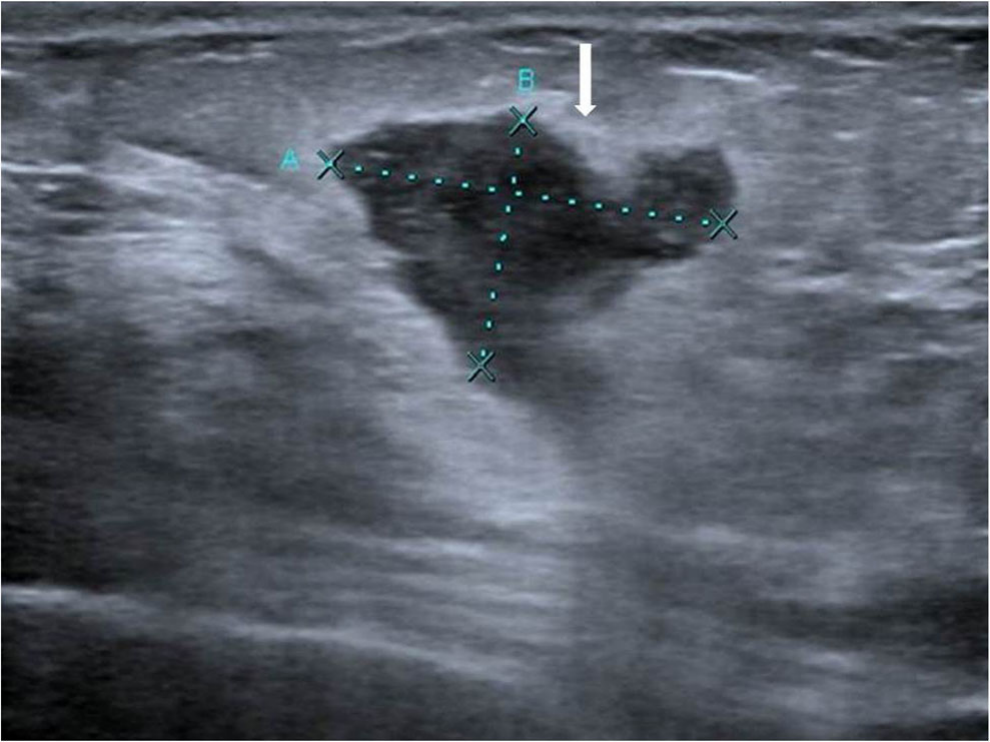
Ultrasound of right breast in a 33-year-old woman presenting with hard painless lump of 3 months duration shows a well-defined hypoechoic lesion with irregular margins. Ultrasound guided biopsy was done and histopathology showed granulomatous mastitis

**Fig. 9:**
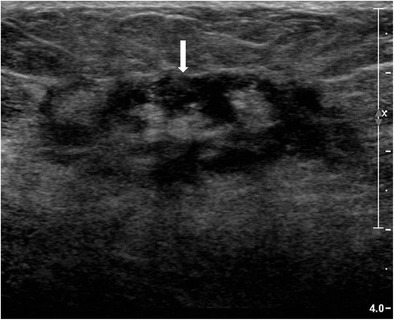
Ultrasound of left breast in a 35-year-old woman presenting with a left breast lump of 1-month duration shows a small ill-defined heterogeneously hypoechoic lesion with central echogenic areas, which was confirmed to be granulomatous mastitis on histopathology

Ultrasound is done using a high frequency (7-10 MHz) linear probe and appearances of this entity are varied. Generally, a hypoechoic or heterogeneous mass(es) is noted, with characteristic tubular hypoechoic extensions connecting the dominant mass to smaller nearby masses (Fig. 7) [13]. Larsen et al., in their study of 54 cases, found an irregular hypoechoic lesion with tubular extension as the most frequent finding [14] and an isolated ill-defined hypoechoic or heterogeneous lesion (Figs. 8 and 9) as the second most common finding. When a mass is detected, colour Doppler examination may be done to assess vascularity.

Parenchymal heterogeneity and distortion with or without acoustic shadowing may also be seen with absence of a definite mass.

**Fig. 10:**
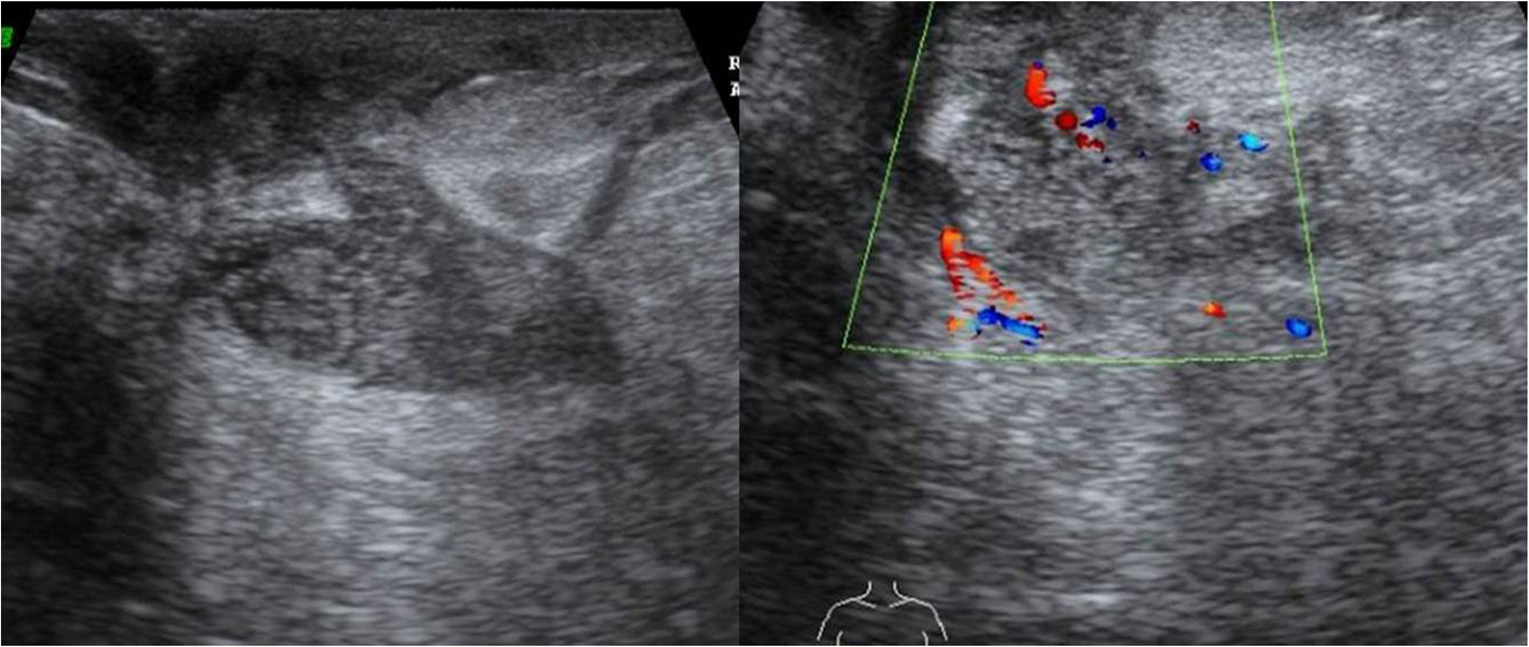
Ultrasound of the right breast from the same patient mentioned in Fig. 1 shows an ill-defined heterogenous lesion with increased vascularity (arrow) and tubular extension (arrowhead). Biopsy was done and histopathology was suggestive of granulomatous mastitis

**Fig. 11:**
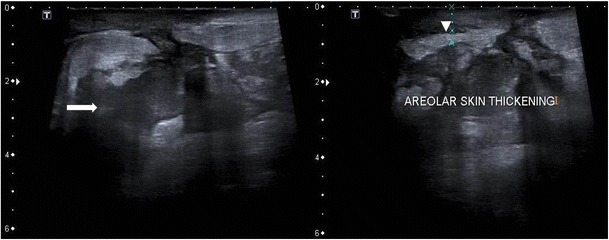
A 32-year-old woman presented with a lump in the left breast of 6-week duration with no history of associated fever. Ultrasound of the left breast showed an ill-defined heterogeneously hypoechoic collection/lesion (arrow) with overlying skin thickening (arrowhead), which was proven to be granulomatous mastitis on histopathology

Sometimes a well-defined collection with low level mobile internal echoes may be present with tubular hypoechoic extension to the subcutaneous tissues and skin (Fig. 10). Associated changes such as overlying skin thickening and nipple retraction can also be seen in a few cases (Fig. 11).

In a study by Kiyak et al. [15], parenchymal heterogeneity, irregular hypoechoic mass, and abscess formation were the most common findings. They opined that parenchymal heterogeneity with abscess formation and axillary lymphadenopathy favor an inflammatory process though histopathology is still essential to establish the diagnosis.

Lee et al. [12] found other synchronous findings such as subcutaneous fat obliteration in almost all cases and skin thickening in 91.7 % cases. Increased surrounding parenchymal vascularity on Doppler ultrasound has also been reported [16].

### The real dilemma in diagnosis

Since the mammographic and ultrasound findings of idiopathic granulomatous mastitis are nonspecific and may often mimic carcinoma, a definitive histopathological diagnosis is essential before contemplating any surgical procedure.

**Fig. 12:**
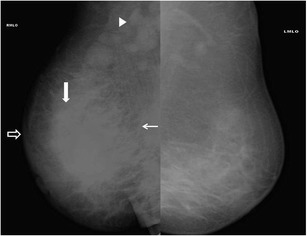
Mammogram (MLO view) of the right breast of a 48-year-old woman presenting with a lump in right breast of 1-month duration shows diffusely increased density in central glandular component (thick arrow) extending to the retromammary space with trabecular thickening (thin arrow). Associated areolar skin thickening (open arrow) and multiple enlarged axillary lymphnodes (arrowhead) are seen. The lesion was proven to be infiltrating ductal carcinoma on histopathology

**Fig. 13:**
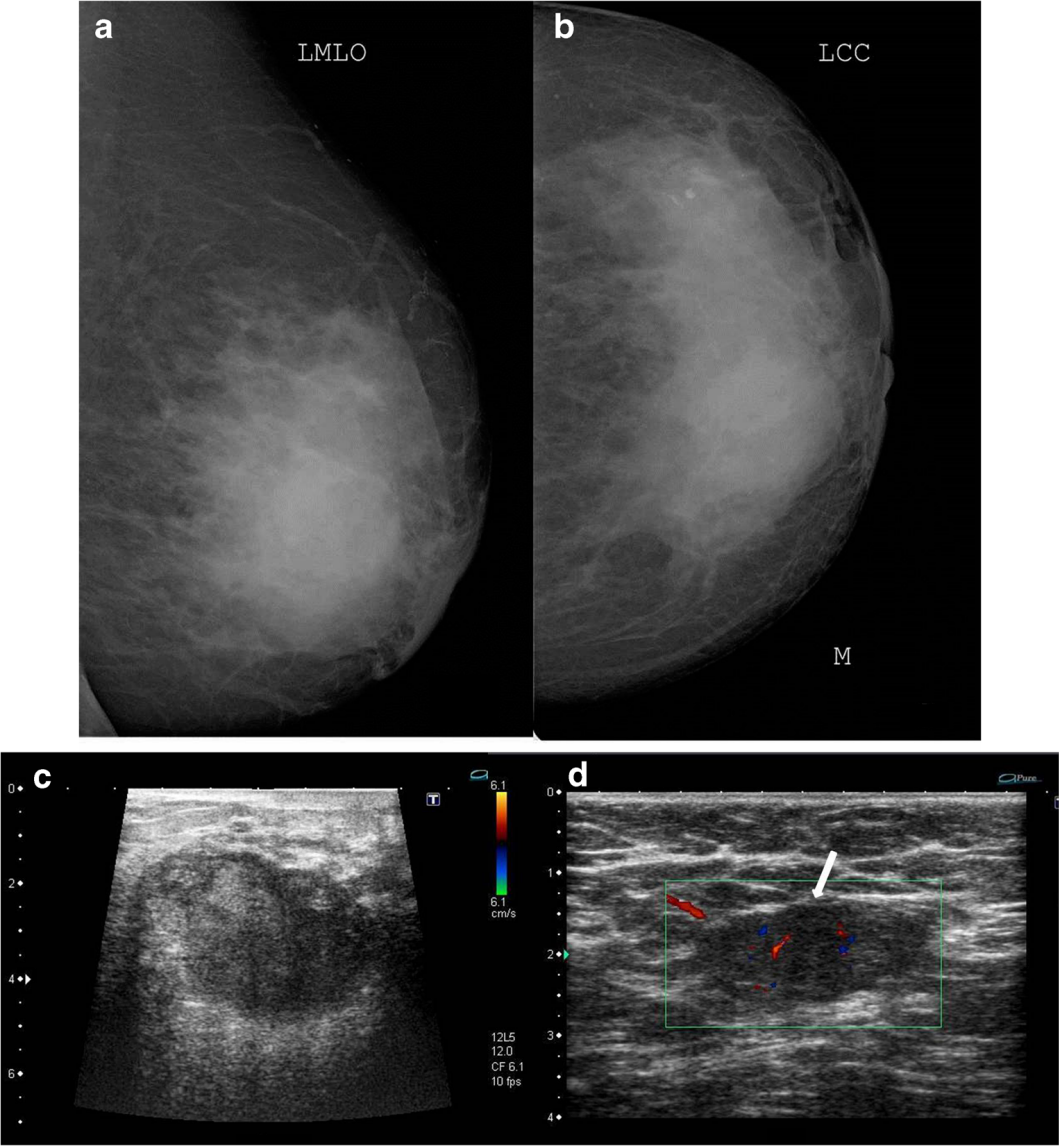
A 42-year-old female presented with swelling and lump in the left breast of 1-month duration. Mammogram (**a**, **b**) showed focal asymmetric density in the lower inner quadrant of the left breast. Ultrasound (**c**) showed a well-defined heterogeneously hypoechoic lesion in retroareolar region with surrounding inflammation extending along the inner quadrant measuring 5 x 3.5 cm in size. The axilla showed enlarged hypoechoic lymph node (**d**) with loss of fatty hilum. Histopathology was s/o idiopathic granulomatous mastitis, and patient was managed conservatively

When there is diffuse involvement of the breast, it is imperative to distinguish idiopathic granulomatous mastitis from inflammatory carcinoma. The most common mammographic finding in the latter is irregular densities with architectural distortion, skin thickening, trabecular prominence due to oedema, and nipple retraction (Fig. 12). Similar mammographic findings may be seen with idiopathic inflammatory mastitis (Fig. 13a and b), which can be confirmed on ultrasound (Fig. 13c, d).

However, the ultrasound feature in favour of benignity is the presence of an ill-defined mass with long axis of lesion parallel to the chest wall. In addition, multifocal abscess cavities, as seen in 28 % cases in a study by Seo et al. [17], may favour a diagnosis of mastitis over malignancy.

Tubercular mastitis, like idiopathic granulomatous mastitis, is also an inflammatory pathology occurring in young parous women and presents with increased breast density with diffuse skin thickening; however, their management differs completely. Seo et al. [17] found that patients with idiopathic granulomatous mastitis were younger, with a mean age at presentation of 33.5 years, as opposed to 40 years in the case of tuberculous mastitis. Also, a higher proportion of patients with idiopathic granulomatous mastitis presented with acute onset mastalgia. The occurrence of axillary lymphadenopathy was more common in tubercular mastitis than idiopathic granulomatous mastitis with an incidence of 50 and 20.6 %, respectively.

On ultrasound, tubercular mastitis has similar imaging features as idiopathic granulomatous mastitis and causes a diagnostic dilemma. It may be seen as a well-defined hypoechoic collection/abscess formation with low level internal echoes or an ill-defined hypoechoic mass with surrounding inflammation. Histopathogical and microbiological confirmation is required to make a definitive diagnosis of tubercular mastitis.

Duct ectasia, periductal mastitis complex, as described earlier, is a result of duct rupture with inflammation in periductal tissues, which may later become infected and cause an abscess formation.

## Role of MRI

Rieber et al. [18] found that MRI did not provide any additional information that was critical in differentiating idiopathic granulomatous mastitis from inflammatory carcinoma, since both exhibit signs of inflammation. In a study by de Bazelaire et al. [19], dynamic contrast MR imaging did not prove to be really discriminatory either, as intense early enhancement (>100 % before 90 s) was found in the majority of cases of inflammatory breast carcinoma and almost half of the patients with mastitis, the enhancement kinetics of both conditions being analogous produced by vascular endothelial growth factor (VEGF). At best, MRI may play a complementary role to increase conspicuity of lesions that are not visualised by mammograms and ultrasound adequately.

## Histopathological evaluation

**Fig. 14:**
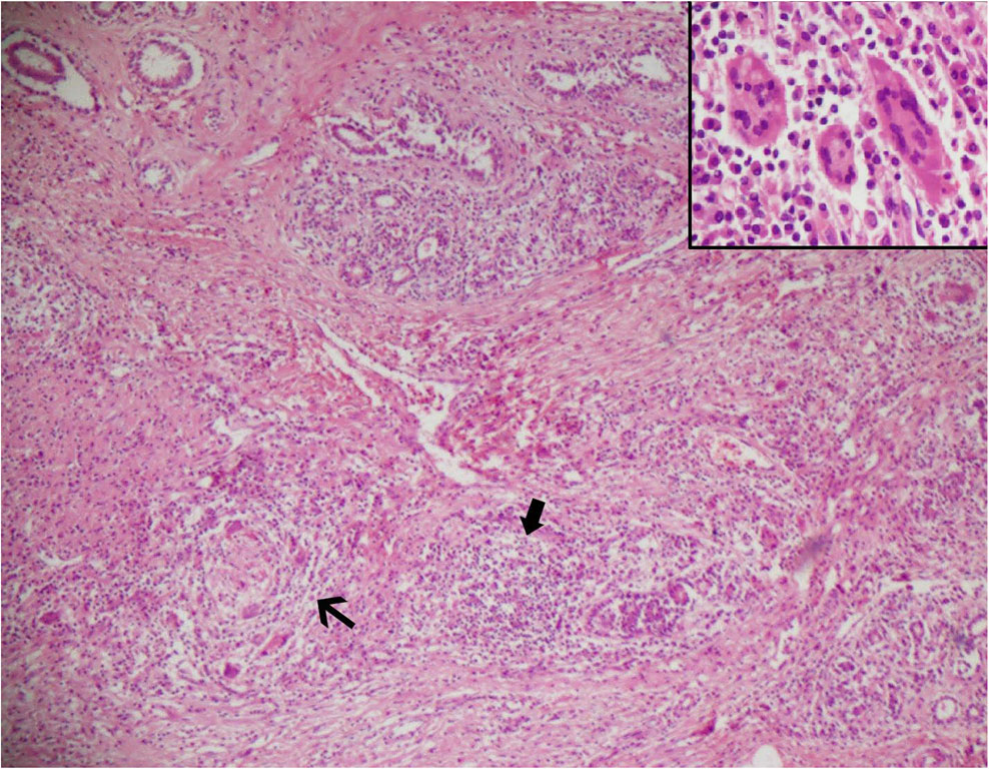
Granulomatous mastitis: 4x magnification showing a forming granuloma (thin arrow) in the background of diffuse lympho-plasmacytic infiltration (thick arrow) of breast parenchyma with scattered giant cells (inset, 40x)

Fine needle aspiration cytology (FNAC) may show polymorphonuclear leucocytes, giant cells, and epitheloid cells (Fig. 14). Histopathology shows chronic lobulitis with non-caseating granulomatous inflammation. In a study of 19 cases by Gangopadhyay et al. [20], caseous necrosis was absent and giant cells were morphologically of foreign body type and Langerhans’s type.

The single most important differential diagnosis of granulomatous mastitis in the Asian sub-continent is tuberculosis. Predominance of neutrophils in the background and relative absence of caseous necrosis favour a diagnosis of granulomatous mastitis. Sometimes these granulomas become confluent and lead to suppuration and liquefactive necrosis. Biopsy should be performed to establish diagnosis before deciding further treatment options.

## Management

The treatment of idiopathic granulomatous mastitis remains controversial, with options ranging from conservative management with antibiotics to wide local excision (WLE) and corticosteroid therapy. Treatment must be tailored to the patient’s clinical presentation. Lai et al. found that spontaneous resolution occurs in 50 % cases in a mean time interval of 14.5 months without any treatment [21]. Kiyak et al. reported that WLE was not the ideal treatment in complicated granulomatous mastitis with abscess formation, fistulas, and diffuse involvement of breast. Most of these patients recovered spontaneously over a mean interval of 5.1 months. They suggested that a short interval follow-up should be done before deciding on steroid treatment.

## Conclusion

1. Idiopathic granulomatous mastitis is a benign entity which has varied and nonspecific appearances on ultrasound and mammography and often mimics malignancy.
2. Mammogram commonly shows focal asymmetric density and skin thickening while parenchymal heterogeneity, irregular mass, and hypoechoic mass with tubular extension are seen on ultrasound.
3. It often mimics breast carcinoma clinically and radiologically, hence histopathological evaluation is necessary to establish diagnosis before deciding upon treatment options.

## References

[1] Kessler E, Wolloch Y (1972). Granulomatous mastitis: a lesion clinically simulating carcinoma. Am J Clin Pathol.

[2] Baslaim MM, Khayat HA, Al-Amoudi SA (2007). Idiopathic granulomatous mastitis: a heterogeneous disease with variable clinical presentation. World J Surg.

[3] Going JJ, Anderson TJ, Wilkinson S, Chetty U (1987). Granulomatous lobular mastitis. J Clin Pathol.

[4] Akcan A, Akyildiz H, Deneme MA, Akgun H, Aritas Y (2006). Granulomatous lobular mastitis: a complex diagnostic and therapeutic problem. World J Surg.

[5] Imoto S, Kitaya T, Kodama T, Hasebe T, Mukai K (1997). Idiopathic granulomatous mastitis: case report and review of the literature. Jpn J Clin Oncol.

[6] Jorgensen MB, Nielsen DM (1992). Diagnosis and treatment of granulomatous mastitis. Am J Med.

[7] Bani-Hani KE, Yaghan RJ, Matalka II, Shatnawi NJ (2004). Idiopathic granulomatous mastitis: time to avoid unnecessary mastectomies. Breast J.

[8] Thomas Stavros A (2004) Breast ultrasound, edition; Colorado: Lippincott William and Wilkins; Chapter 11, Non-malignant breast disorders that have complex cystic phases 372–374

[9] Yilmaz E, Lebe B, Usal C, Balci P (2001). Mammographic and sonographic findings in the diagnosis of idiopathic granulomatous mastitis. Eur Radiol.

[10] Memis A, Bilgen I, Ustun EE, Ozdemir N, Erhan Y, Kapkac M (2002). Granulomatous mastitis: imaging findings with histopathologic correlation. Clin Radiol.

[11] Han BK, Choe YH, Park JM, Moon WK, Ko YH, Yang JH (1999). Granulomatous mastitis: mammographic and sonographic appearances. AJR Am J Roentgenol.

[12] Lee JH, Oh KK, Kim EK, Jung WH, Kwack KS, Lee HK (2006). Radiologic and clinical features of idiopathic granulomatous lobular mastitis mimicking advanced breast cancer. Yonsei Med J.

[13] Yeong Yi A (2011). Diffuse infiltrative lesion of the breast: clinical and radiologic features. Korean J Radiol.

[14] Hovanessian Larsen LJ, Peyvandi B, Klipfel N, Grant E (2009). GeetaIyengar, granulomatous lobular mastitis- imaging, diagnosis and treatment. AJR.

[15] Kiyak G, Dumlu EG, Kilinc I, Tokaç M, Akbaba S, Gurer A (2014). Management of idiopathic granulomatous mastitis: dilemmas in diagnosis and treatment. BMC Surg.

[16] Boufettal H, Essodegui F, Noun M, Hermas S, Samouh N (2012). Idiopathic granulomatous mastitis: a report of twentycases. Diagn Interv Imaging.

[17] Seo HRN, Na KY, Yim HE (2012). Differential diagnosis in idiopathic granulomatous mastitis and tuberculous mastitis. J Breast Cancer.

[18] Rieber A, Tomczak RJ, Mergo PJ, Wenzel V, Zeitler H, Brambs HJ (1997). MRI of the breast in the differential diagnosis of mastitis versus inflammatory carcinoma and follow-up. J Comput Assist Tomogr.

[19] de Bazelaire C, Groheux D, Chapellier M, Sabatier F, Scémama A, Pluvinage A (2012). Breast inflammation: indications for MRI and PET-CT. Diagn Interv Imaging.

[20] Gangopadhyay et al (2010) The diagnosis of the idiopathic granulomatouz mastitis. J Turkish German Gynecol Assoc 11: 127–130 10.5152/jtgga.2010.18 PMC3939218 24591917

[21] Lai EC, Chan WC, Ma TK, Tang AP, Poon CS, Leong HT (2005). The role of conservative treatment in idiopathic granulomatous mastitis. Breast J.

